# A Rare Case of an Adrenal Mass Proved to Be a Benign Cellular Schwannoma

**DOI:** 10.7759/cureus.23296

**Published:** 2022-03-18

**Authors:** Ilias Galanis, Georgios Floros

**Affiliations:** 1 Department of Surgery, Evangelismos General Hospital, Athens, GRC

**Keywords:** adrenalectomy, cellular schwannoma, retroperitoneal tumor, adrenal schwannoma, adrenal mass

## Abstract

Adrenal schwannomas are extremely uncommon tumors. We report the case of a 39-year-old male with a right adrenal mass. Laboratory tests were normal and radiological exams revealed the adrenal tumor. Open surgical adrenalectomy was performed. The postoperative course was uneventful. Microscopy and immunohistochemistry revealed a cellular schwannoma. Adrenal schwannomas are rare tumors, very difficult to diagnose in preoperative evaluation. Surgical excision of the tumor, histological and immunohistochemical examination of the specimen provide a definitive diagnosis. Prognosis is generally very good. Recurrence rates are related to positive surgical margins.

## Introduction

Schwannomas are infrequent tumors deriving from Schwann cells of peripheral nerves sheaths. They were first described by Jose Verocay in 1908, and in 1920, they were subclassed by Antoni into two histologic patterns [1]. Schwannomas are most often found within the head, neck, upper and lower extremities. They may, rarely, be reported in the posterior mediastinum and the retroperitoneum [2]. Retroperitoneal schwannomas account for 3% of all schwannomas [3]. They usually arise between the second and the fifth decade of life, while their distribution in terms of sex is controversial, with some studies reporting a female predominance, while others note that there is no predisposition for sex [4]. Adrenal schwannoma is a rare type of retroperitoneal schwannoma, with fewer than 60 cases reported. It is thought to originate either from the sympathetic nerves from the upper lumbar plexus or the phrenic or vagus nerves, which are nerve complexes with myelin sheaths innervating the adrenal medulla [5]. For this reason, these tumors have been revealed to arise from the adrenal medulla, compressing, in most cases, the adrenal cortex around them [6].

## Case presentation

**Figure 1:**
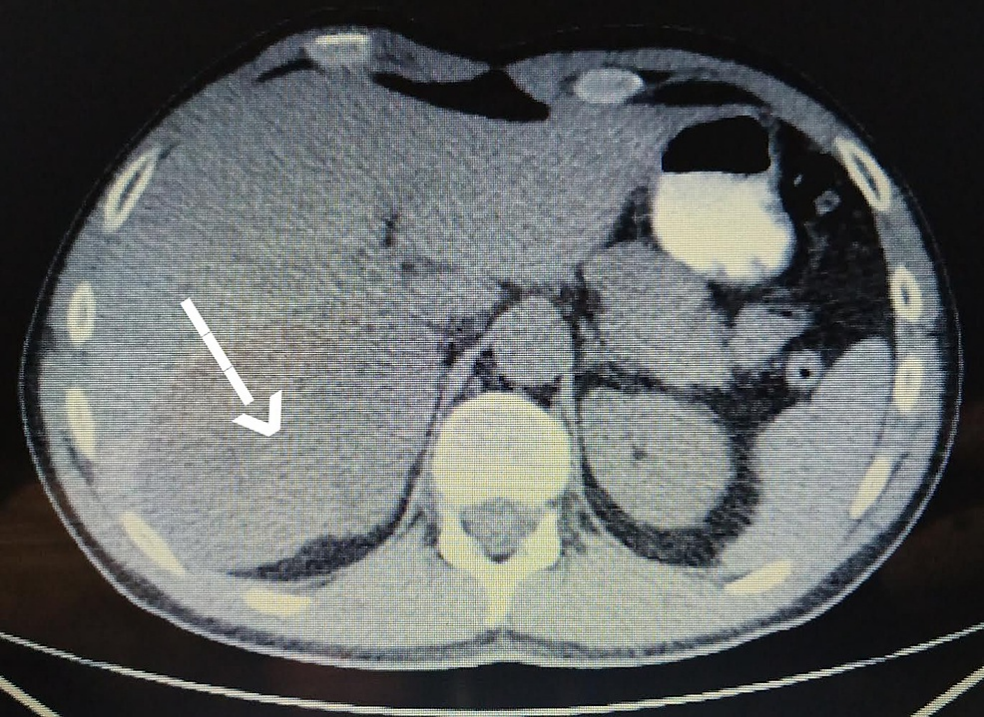
CT scan indicating an adrenal mass

**Figure 2:**
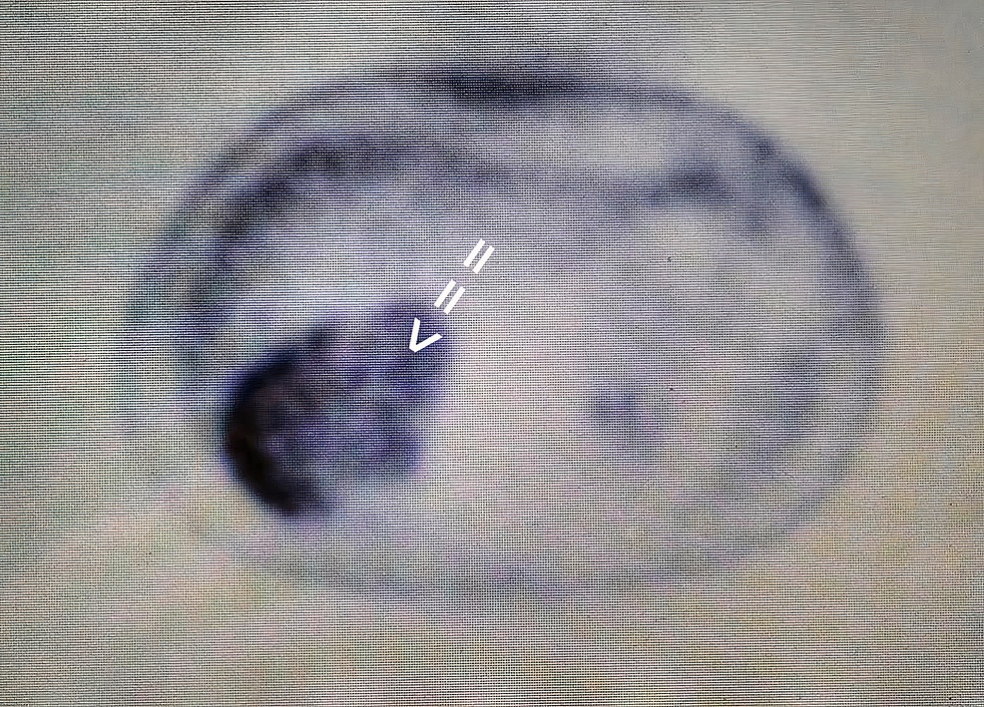
Intense uptake within the right adrenal nodule in fluorodeoxyglucose positron emission tomography (FDG-PET)

A 39-year-old male was referred to the department of endocrinology of our hospital for further investigation of a 12x8.5 cm right adrenal mass, which was found on abdominal ultrasonography performed due to abdominal pain. Past medical history included syncopal episodes, which were investigated by our department of cardiology. A magnetic resonance imaging (MRI) of the retroperitoneal space revealed a large solid adrenal mass of 12x8.5 cm with increased T2 signal intensity and decreased T1 signal intensity. The tumor compressed the liver, the right kidney, and the inferior vena cava without presenting an invasive trend. The tumor was suspicious for pheochromocytoma. However, all endocrinological examinations were within the normal range, and the metaiodobenzylguanidine (MIBG) scan was negative. A fluorodeoxyglucose positron emission tomography (FDG-PET/CT) was also done, indicating high uptake in the right adrenal gland, with a standardized uptake value (SUVmax) of 9.5 (Figures 1-2).

**Figure 3:**
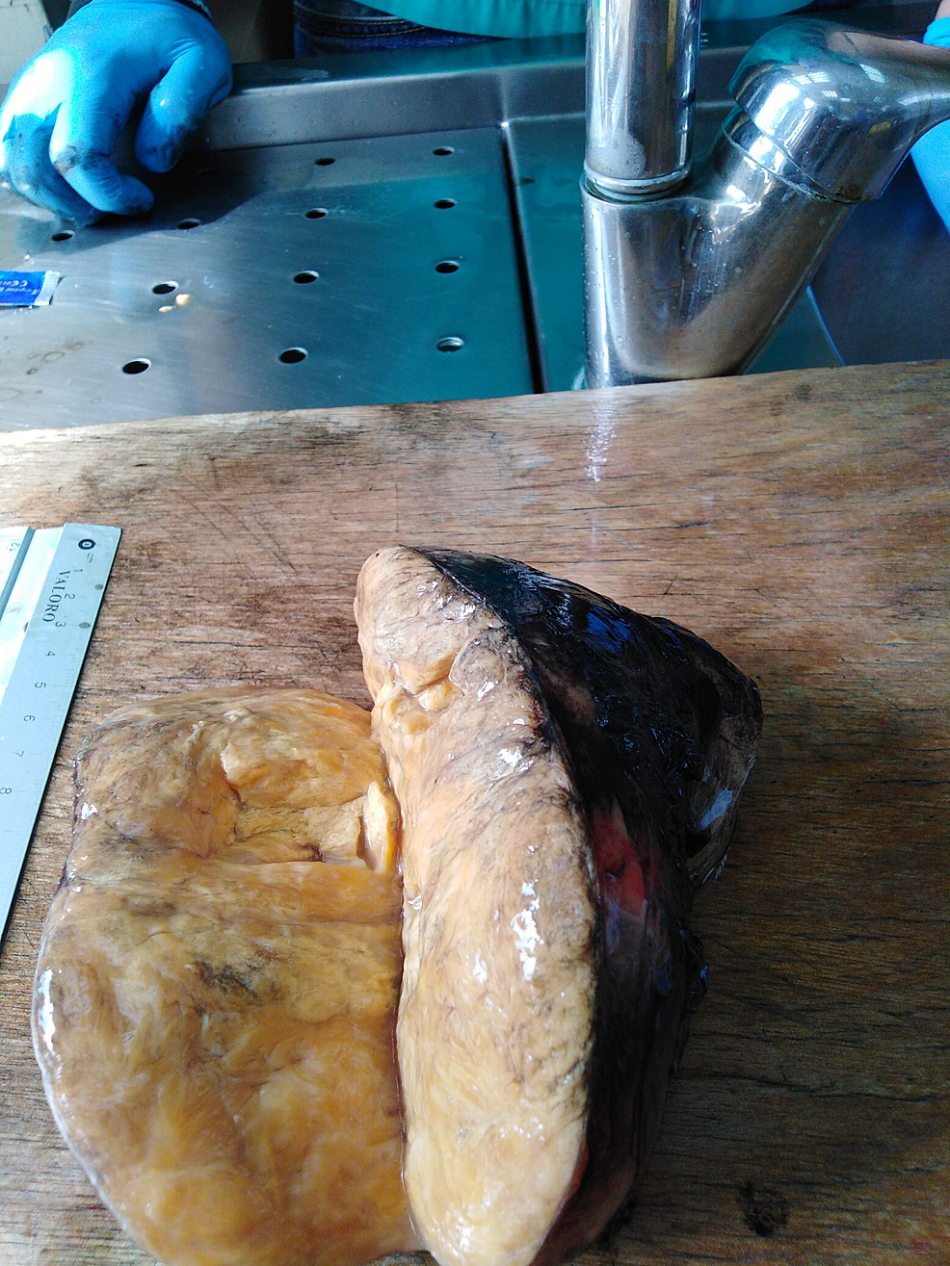
Adrenal mass before the histological examination

The patient was then referred to our surgery department for further evaluation. Because of the size of the mass, open right adrenalectomy was performed without complications. A well-circumscribed encapsulated solid mass grossly compressing the adrenal parenchyma, 11.5x9x5.5 cm in dimensions, was discovered (Figure 3).

**Figure 4:**
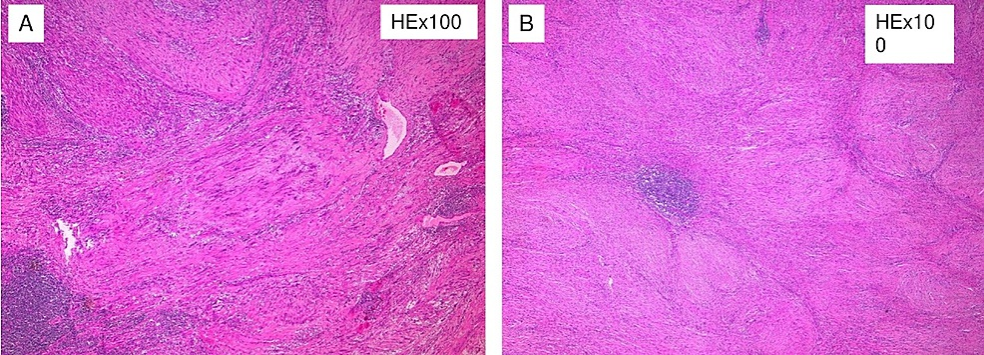
The tumor is composed of spindle cells arranged in cellular (Antoni A) and nodular fascicular pattern

**Figure 5:**
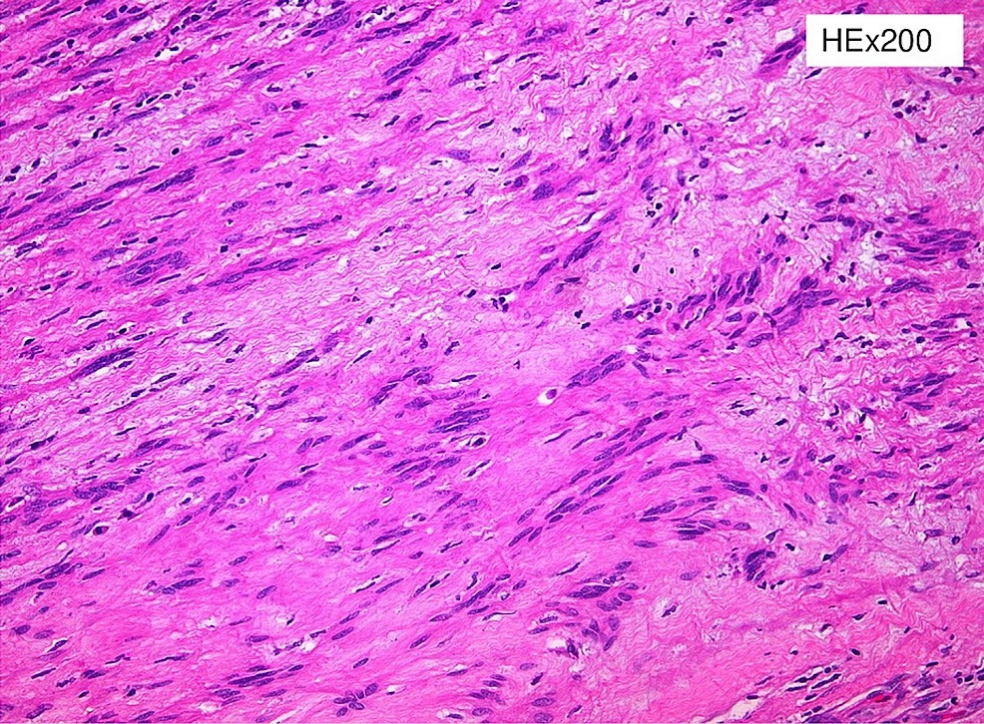
Verocay bodies

**Figure 6:**
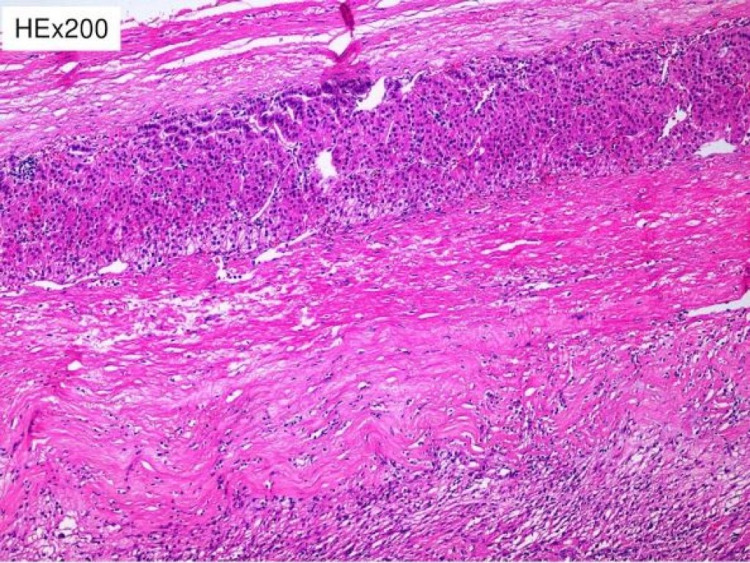
Residual adrenal cortex is recognized at the periphery of the tumor

**Figure 7:**
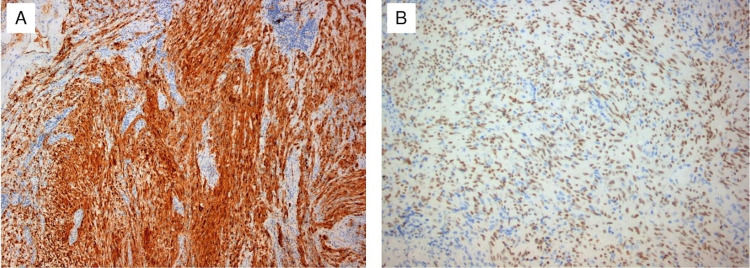
Diffuse and strong expression of S-100 (A) and strong nuclear expression of SOX-10 (B)

Histologically, the tumor was a benign cellular schwannoma, consisting mainly of areas with high cellularity (Antoni A) and very few areas with low cellularity (Antoni B; Figure 4). A few Verocay bodies were also present (see Figure 5). The tumor compressed the adrenal gland without invading it (Figure 6). Immunohistochemical analysis demonstrated diffusely positive S-100 and SOX-10 staining across the tumor (Figure 7), very low mitotic rate (three mitoses/ 10 high power fields), and Ki-67 proliferation index positive in 6% of cells. Fluorescence in situ hybridization (FISH) revealed homozygous deletion of CDKN2A in 5% of nuclei, heterozygous deletion of CDKN2A in 29% of nuclei, and chromosome 9 monosomy in 44%. Surgical margins were negative. The patient recovered well after surgery and was discharged on the sixth postoperative day. An ultrasound of the abdomen was recommended three months after surgery and a CT six months after surgery.

## Discussion

Schwannomas are usually benign [7]. Ninety percent of cases are sporadic, while 3% are associated with Von Recklinghausen disease. When they are spotted in the retroperitoneal region, 5-18% of schwannomas are related to Von Recklinghausen disease [8]. In these cases, they present in atypical locations and have a greater malignant tendency. While benign tumors only displace structures and organs surrounding them, malignant schwannomas present an invasive growth pattern with a tendency to develop distant metastases [6].

Retroperitoneum is a large and flexible space. Therefore, detection of retroperitoneal schwannomas is often incidental, as they have to be very large in size before they become symptomatic. In late stages, large tumor signs present with lumbar or abdominal pain or distension, neuralgia or paresthesia, and obstruction of the ureters [9]. Large adrenal schwannomas may also compress the inferior vena cava, as in our case, or other surrounding structures.

Pre-operative diagnosis of adrenal schwannomas is difficult as this tumor has no special features in computed tomography (CT) or MRI [5]. CT usually shows a well-defined mass with cystic necrotic central areas and calcification. MRI findings include low signal intensity in T1 weighted image (T1WI) and heterogeneously high intensity in T2 weighted image (T2WI) for solid tumors. Benign adrenal masses may be discriminated from malignant ones with an FDG-PET, which presents a sensitivity between 93% and 100% and a specificity between 80% and 100% [10]. Metabolic evaluation of the tumor with endocrinological examinations, including serum electrolytes, cortisol, urinary metanephrines, adrenalin, noradrenalin, vanillyl mandelic acid (VMA) as well as renin and aldosterone, must be conducted. Fine needle aspiration or fine needle biopsy is not recommended because of the risks of hemorrhage, infection, seeding of tumor cells, and the consequences of a possible pheochromocytoma existence [5]. Definitive diagnosis is only made by histological and immunohistochemical examination of the mass. Two distinct patterns have been described: Antoni A hypercellular areas with nuclear palisades and Verocay bodies, which are nuclear-free zones between the regions of nuclear palisading, and Antoni B paucicellular areas. These two patterns may coexist, but usually, one is predominant. Immunohistochemical staining shows tumor cells positive for S-100 protein, SOX-10, and vimentin [1].

The appropriate treatment for adrenal schwannoma is complete excision, as these tumors do not present sensitivity neither to chemotherapy nor to radiotherapy [11]. The surgical approach remains debatable. Both laparoscopic and open surgery may be used, but in schwannomas and every adrenal mass larger than 5 cm, open surgery is believed to be the technique of choice. In our case, due to the large size of the tumor, which not only creates technical difficulties on surgery but also maximizes the chance of malignancy, we decided that open adrenalectomy was the suitable treatment choice. Our department's size threshold for open adrenalectomy is 5 cm. The matter of negative soft tissue margins remains controversial. Some argue that because malignancy cannot be excluded preoperatively and because the local recurrence rate ranges, negative margins are necessary. Others support that as it is usually a benign mass, a simple enucleation or partial excision is sufficient [12]. The prognosis of adrenal schwannoma is extremely good. Reported recurrence (5-10%) is due to incomplete excision, and surgical resection of the lesions is recommended [3]. Periodic follow-up of patients with control imaging studies, such as CT or MRI, is suggested bi-annually for two years and once every year for three more years [13].

## Conclusions

Adrenal schwannoma is a rare, benign tumor with a very good prognosis. Its diagnosis is based on histopathology and immunochemistry, and surgical excision remains the treatment of choice. Physicians should be aware of schwannomas so that they may suspect them in any case of a retroperitoneal adrenal mass.
